# Surface Modified β-Tricalcium phosphate enhanced stem cell osteogenic differentiation in vitro and bone regeneration in vivo

**DOI:** 10.1038/s41598-021-88402-5

**Published:** 2021-04-29

**Authors:** Cheuk Sing Choy, Wei Fang Lee, Pei Ying Lin, Yi-Fan Wu, Haw-Ming Huang, Nai-Chia Teng, Yu-Hwa Pan, Eisner Salamanca, Wei-Jen Chang

**Affiliations:** 1 Department of Community Medicine, En Chu Kong Hospital, New Taipei City, Taiwan; 2 Yuanpei University of Medical Technology, Hsinchu, Taiwan; 3 School of Dental Technology, College of Oral Medicine, Taipei Medical University, Taipei, Taiwan; 4 School of Dentistry, College of Oral Medicine, Taipei Medical University, Taipei, Taiwan; 5 Graduate Institute of Biomedical Materials and Tissue Engineering, College of Oral Medicine, Taipei Medical University, Taipei, Taiwan; 6 Dental Department, Taipei Medical University Hospital, Taipei, Taiwan; 7 Department of General Dentistry, Chang Gung Memorial Hospital, Taipei, Taiwan; 8 Graduate Institute of Dental and Craniofacial Science, Chang Gung University, Taoyuan, Taiwan; 9 School of Dentistry, College of Medicine, China Medical University, Taichung, Taiwan; 10 Dental Department, Taipei Medical University, Shuang-Ho hospital, Taipei, Taiwan

**Keywords:** Stem-cell research, Biomedical engineering, Implants

## Abstract

A major number of studies have demonstrated Beta-tricalcium phosphate (β-TCP) biocompatibility, bioactivity, and osteoconductivity characteristics in bone regeneration. The aim of this research was to enhance β-TCP's biocompatibility, and evaluate its physicochemical properties by argon glow discharge plasma (GDP) plasma surface treatment without modifying its surface. Treated β-TCP was analyzed by scanning electron microscopy (SEM), energy-dispersive spectrometry, X-ray photoelectron spectroscopy (XPS), X-ray diffraction analysis, and Fourier transform infrared spectroscopy characterization. To evaluate treated β-TCP biocompatibility and osteoblastic differentiation, water-soluble tetrazolium salts-1 (WST-1), immunofluorescence, alkaline phosphatase (ALP) assay, and quantitative real-time polymerase chain reaction (QPCR) were done using human mesenchymal stem cells (hMSCs). The results indicated a slight enhancement of the β-TCP by GDP sputtering, which resulted in a higher Ca/P ratio (2.05) than the control. Furthermore, when compared with control β-TCP, we observed an improvement of WST-1 on all days (*p* < 0.05) as well as of ALP activity (day 7, *p* < 0.05), with up-regulation of ALP, osteocalcin, and Osteoprotegerin osteogenic genes in cells cultured with the treated β-TCP. XPS and SEM results indicated that treated β-TCP’s surface was not modified. In vivo, micro-computed tomography and histomorphometric analysis indicated that the β-TCP test managed to regenerate more new bone than the untreated β-TCP and control defects at 8 weeks (*p* < 0.05). Argon GDP treatment is a viable method for removing macro and micro particles of < 7 μm in size from β-TCP bigger particles surfaces and therefore improving its biocompatibility with slight surface roughness modification, enhancing hMSCs proliferation, osteoblastic differentiation, and stimulating more new bone formation.

## Introduction

Autografts are still considered the gold standard for the treatment of defects and constitute more than half of the bone grafts used^1^. Techniques using this material suffer multiple drawbacks, including a short supply of the required materials and the need for additional bone extraction surgery, which leads to donor-site morbidity^1^. To circumvent these drawbacks, researchers have developed a pure-phase beta-tricalcium phosphate (β-TCP) alternative to patient's own tissue. This material is highly biocompatible and bioresorbable and facilitates new bone formation. It has an intragranular porosity of 65%, and the granulates (i.e., polygonal morsels) particle size between 150 and 500 μm or from 500 to 1000 μm. Because of its consistent porosity and calcium-to-phosphorus ratio, β-TCP provides predictable resorption and new bone formation within 4–12 months. Within β-TCP granules, the capillary effect of the blood promotes the rapid formation of osteoblasts, which stimulates vital bone growth^2^.

β-TCP has become the preferred option in multiple dental procedures. A previous study about β-TCP showed improvements in new bone formation in accelerated osteogenic orthodontics^3^. Moreover, a previous research investigated the effect of β-TCP combined with a limited amount of particulate autogenous bone, mixed in equal ratios, in newly bone regeneration procedure after maxillary sinus augmentation^4^. These studies did not find any difference between the use of autogenous bone alone or in combination with β-TCP when measuring new bone while clinically reducing donor-site morbidity^4^. Some β-TCP properties, including its inflexible structure and osteoconductive nature, both of which can facilitate bone development, are ideal for treating larger, more critical bone defects. Clinically, the application of β-TCP in orthopedic impairments and minor localized bone defects around the teeth is limited^5^. Several studies have assessed the utilization of β-TCP bioceramic for bone tissue regeneration. Nonetheless, its disadvantages including limited bone formation should be addressed^6^.

Previous studies have found that glow discharge plasma (GDP) can be used widely for cleaning, etching, and polymerization on biomaterial surfaces^7–9^. Studies have demonstrated that appropriate argon GDP conditions are a powerful tool for modifying surfaces without leaving a trace of free, potentially problematic entities for biomaterial surfaces, which results in improved tissue regeneration outcomes. It has been demonstrated previously that cell responses during guided bone regeneration can be mediated by surface roughness and chemical composition^10^. Impurities can affect the chemical characteristics of β-TCP, and they are challenging to control while producing alloplast. Furthermore, in cases of mass production, simple line productions are valuable due to commercial reasons.

Hence, further investigation about plasma-assisted techniques for fabricating β-TCP surface-treated GDP biomaterial should be conducted to evaluate their ability to increase surface biocompatibility while not affecting, or perhaps even lowering, their toxicity^11,12^. Such surface treatments could stimulate wanted reactions from the bone graft particles surrounding tissues and increase bone regeneration. The present research aimed to enhance β-TCP's biocompatibility, and evaluate its physicochemical properties by argon glow discharge plasma (GDP) plasma surface treatment without surface modification.

## Materials and methods

### In vitro

#### Preparation of β-TCP

We used pure-phase β-TCP with a granulation size of 500–1000 µm, 65% ± 5% total porosity, 20% 5–50-µm, and 15% 50–200-µm pore diameter^13^ (CERASORB M, Curasan Co Ltd, Frankfurt, Germany); 200 mg control samples were prepared, with the same amount used for GDP treatment.

#### Gas discharge plasma treatment

We used the plasma jet device (AST Products Inc., North Billerica, MA, USA) for the argon GDP of the β-TCP test particles. The GDP treatment was set at 80 W using a radiofrequency of 13.56 MHz and 100 mTorr working pressure. The treatment time was 15 min with 10 mm argon plasma working distance from the β-TCP particles.

#### Surface morphological characterization

To analyze the surface morphologies of the scaffolds, β-TCP particles treated with argon gas discharge plasma were analyzed and compared with nontreated β-TCP. We used scanning electron microscopy (SEM; EX-250 SYSTEM, HORIBA, Kyoto, Japan) images to observe the microstructure and crystal size of the particles. These images were analyzed using ImageJ version 1.52 (NIH IMAGE, Bethesda, MD, USA). The arithmetical mean value roughness (Ra) was calculated for quantitative analyses.

#### Energy-dispersive spectrometry

For particle’s elements qualitative and quantitative analysis, energy-dispersive X-ray spectroscopy (EDS) was conducted. β-TCP test and control samples elemental composition analysis were performed using the same SEM. For the surface morphology evaluation, a machine that combines SEM and EDS was used (EX-250 SYSTEM, HORIBA, Kyoto, Japan).

#### X-ray photoelectron spectroscopy

Chemical analyses were achieved by a surface X-ray photoelectron spectroscopy (XPS) analysis technique with a depth profiling of approximately 50–70 Å from the surface using a monochromated 450 W Al Kα source (PERKIN-ELMER PHI ESCA 5500 SYSTEM). 220-W source power and 45° analyzer axis with angular acceptance of ± 7° were used for experiments recording. The charging shift was referred to the C1s line emitted from the saturated hydrocarbon at a binding energy of 285 eV. We recorded information on the chemical state of the core levels of the detected elements C1s, C1s, O1s, Ca2p, and P2p^14^.

#### X-ray diffraction analysis

β-TCP test and β-TCP control particles crystalline structures and chemical compositions were analyzed using powder X-ray diffraction (XRD). Pattern analyses were performed at a 60-kV and 45-mA current with Mo Kα λ = 0.71073 Å source. A diffractometer was used for all analyses in the range of 10° ≤ 2θ ≤ 70° (XPERT3 PRO, PANalytical Co. Ltd., Almelo, the Netherlands).

#### Fourier transform infrared spectroscopy

We measured 0.2-g powdered sample spectra from both the dry β-TCP test and β-TCP control on a Fourier transform infrared spectrometer (NICOLET iS50, Thermo Scientific, Madison, USA). Measurements were made in the wavelength range 4000–400 cm^−1^ with a resolution of 4 cm^−1^ at 25 °C and 65% ± 5% humidity. Three spectra were collected for each sample in the absorbance mode, including subtraction of a background scan, in order to reduce noise. Thus, the average of the three measurements were averaged to produce one spectrum^15^.

#### Cell culture

Human mesenchymal stem cells (hMSCs) were acquired from the Bioresource Collection and Research Center (Hsinchu, Taiwan). Moreover, they were maintained at 37 °C in humidified incubators with 5% CO_2_/95% air in specific culture media based on the protocol of our previous study, as described below^16^. Briefly, hMSCs were cultured in Dulbecco’s modified Eagle’s medium (DMEM; HyClone, Logan, UT, USA) supplemented with l-glutamine (4 mmol/L), 10% fetal bovine serum, and 1% penicillin–streptomycin. The confluent cells were expanded until passage 3, using 0.05% trypsin–EDTA. The final concentration was adjusted to 1 × 10^4^ cells/mL, and aliquoted into 24-well Petri dishes (NUNCLON; Roskilde, Denmark). On the same day, DMEM was mixed with β-TCP control or β-TCP plasma treated at a concentration of 1 g/10 mL. Twenty-four hours later, the medium was removed from each test well and substituted for the test media, consisting of the previously described DMEM + β-TCP control or β-TCP plasma treated. Same Dulbecco’s modified Eagle’s medium first described was used on control wells.

#### Cell viability (WST-1)

Cell viability was measured at days 1, 3, and 7 after adding DMEM + β-TCP control or β-TCP plasma treated to the test wells. Cell viability was measured using a colorimetric assay for 96-well plates with 2-(4-iodophenyl)-3-(4-nitrophenyl)-5-(2,4-disulfophenyl)-2H-tetrazolium monosodium salt reagent (WST-1 Kit, Roche Applied Science, Mannheim, Germany). Summarily, the cell medium was replaced with 500 µL fresh medium, and 100 µl were added into 96-well microtiter plate (5 × 10^4^ cells/well) and incubated for 24 h. Later a 10 µl of cell proliferation reagent WST-1 was added to each well and incubated for 2 h. Cell viability was measured at 450 nm in an ELISA reader (Thermo Fisher Scientific Inc., USA) with a reference wavelength of 650 nm. The percentage viability was calculated from the following equation: % viability = (100 × (control − sample))/control^17^.

#### Immunofluorescence

The hMSCs were prepared for immunofluorescence microscopy on days 1, 3, and 7, in 24-well Petri dishes (NUNCLON; Roskilde, Denmark) as previously described in cell culture. The hMSCs were washed using 2× phosphate-buffered saline (PBS) and fixed in 4% paraformaldehyde for 15 min. After fixation cells were washed three times with PBS for 10 min. Cells were permeabilized using 0.2% Triton X-100 for 20 min, washed three more times, blocked with 1% goat serum in PBS for 1 h, and incubated overnight with primary antibodies in 0.1% goat serum at 4 °C. The cells were washed three times and incubated with secondary antibodies in 0.1% goat serum for 2 h at ambient temperature. After 3× washes, samples were quenched with 0.5% (wt/vol) Sudan Black B (Sigma-Aldrich, St. Louis, MO, USA) for 10 min, nuclear counterstaining was performed using 300 μl of DAPI (0.1 μg/mL) for 10 min. When phalloidin staining was performed, 200 μl of phalloidin solution (methanol-based stock solution diluted in PBS once to obtain a 100-μl final concentration) was added for 15 min after staining with the secondary antibodies and two washing steps with PBS. Residual phalloidin removal was performed before mounting. The same methodology was utilized for three-dimensional (3D) immunofluorescence microscopy after cells were cultured in TCP control or β-TCP plasma-treated particles for 24 h. The immunofluorescent-labeled samples were placed on glass slides and viewed using the Olympus Fluoview FV-1000 confocal laser-scanning microscope (Olympus, Japan), equipped with a 40× oil objective. The fluorescence images from DAPI and Phalloidin were merged using Leica LAS X software.

#### Alkaline phosphatase assay

Alkaline phosphatase (ALP) activity was determined by modifying the previously reported methods^16^. After cell culture media was suctioned and DMEM + β-TCP control or β-TCP plasma treated media were added to the test wells. On days 1, 3, and 7, hMSCs were washed twice with PBS and resuspended in 300 μl of Triton-100 0.05%. The cells underwent three cycles of 5 min at 37 °C and 5 min at − 4 °C. Afterward, using the Thermo Scientific 1-Step p-nitrophenyl phosphate disodium salt (PNPP) protocol. 100 µL of the 1-Step PNPP was added to each 96-well plate and gently mixed. Following incubation at room temperature for 30 min. Next, the reaction was stopped by adding 0.4 M of NaOH, and the plate was read at a wavelength of 405 nm in the Multiskan GO microplate spectrophotometer assay reader (Thermo Fisher Scientific).

#### Real-time polymerase chain reaction

The cultures of hMSCs were completed on days 0 (only for control), 1, 3, and 7, as previously described, in cell cultures after the media were suctioned, and DMEM + β-TCP control or β-TCP plasma-treated media were added to the test wells. Total RNA was extracted using the Novel Total RNA Mini Kit (NOVELGENE, Molecular Biotech, Taiwan) under the conditions recommended by the manufacturer. The cells were trypsinized, harvested, and resuspended; subjected to cell lysis and RNA binding; and washed and eluted, as previously described^18,19^.

Subsequently, gene expression levels were normalized to the expression of the housekeeping gene glyceraldehyde 3-phosphate dehydrogenase. The analysis results were expressed as time-course gene changes relative to the cell's genes cultured in DMEM only, and the calibrator sample representing the amount of transcript, was expressed on day 0^20^. After the design of multiple primers, ALP, OC, CatK, Rank, RankL, OPG using the Primer-BLAST from the U.S. National Library of Medicine. Reactions were run using 2 μl of cDNA in a 20-μL reaction volume on LightCycler 96 system (Roche Molecular Systems, Inc., Pleasanton, CA, USA) with Fast SYBR Green Master Mix (Thermo Fisher Scientific, Vilnius, Lithuania) (Table 1). The reaction was repeated for 45 cycles; each cycle consisted of denaturing at 95 °C for 15 s and annealing, synthesis at 60 °C for 1 min and extension at 72 °C for 30 s, as per the manufacturer’s instructions. The relative amounts of the transcript of the tested genes were normalized using a human glyceraldehyde-3-phosphate dehydrogenase (GAPDH). Posterior quantification was performed using the delta–delta calculation method^16,21^.

**Table 1:** Primer sequences for real time polymerase Chain reaction.

Gene symbol	Forward primer sequence (5′ > 3′)	Reverse primer sequence (5′ > 3′)
ALP	AGCCTTCCTGAAAGAGGATTGG	GCCAGTACTTGGGGTCTTTCT
OC	TCCTTTGGGGTTTGGCCTAC	CCAGCCTCCAGCACTGTTTA
RANKL	ACTGGCCTCTCACCTTTTCTG	AGCCATCCACCATCGCTTTC
CatK	ACCCACGGGAAGCAGTACAA	GGCCTCAAGATTATGGACAGAAA
OPG	CTGGAACCCCAGAGCGAAAT	GCCTCCTCACACAGGGTAAC
RANK	GAAGGTGGACTGGCTACCAC	TTTCCTTCCCCTCCCCAGAA
GAPDH	CCTCCTGTTCGACAGTCAGC	CCTAGCCTCCCGGGTTTCTC

### In vivo analysis

#### Surgical procedure

This in vivo animal experiment was conducted based on the ARRIVE guidelines^22^. The Experimental Animal Research of the Institutional Animal Care and Use Committee (IACUC) of Master Laboratory Co., Ltd. (IACUC no.: MI-201903–02, Hsinchu County, Taiwan) approved all experiments and animal care procedures. All surgical procedures were performed in accordance with the Animal Research: Reporting In Vivo Experiments guidelines. 15 adult male New Zealand white rabbits with a mean age of 3 months and a mean weight of 2.1 kg were ultimately enrolled in the present study. The animals were housed in separated cages in a climate-controlled Laboratory Animal Center, and the animals had ad libitum access to food and water.

Following an intramuscular anesthesia injection of Zoletil 50 (50 mg/mL) into the gluteal region at a dose of 15 mg/kg, the calvarial region was shaved and disinfected with iodine. Next, on the periphery of the calvaria for hemostatic and local anesthetic, 1.8 mL of 2% lidocaine with epinephrine 1/100,000 was injected.

A 2-cm long full depth incision was made in the *linea media* of the calvaria starting midway between the base of the ears. The pericranium was separated with a periosteal elevator from the outer table of the cranial vault^23^.

To prevent brain damage, the parietal bone was perforated three times using a 6.0-mm sterile trephine (3I Implant Innovation, Palm Beach Gardens, FL, USA)^24^. The first defect was filled with β-TCP control, and the second with β-TCP plasma-treated media. The third defect was left to heal as an unfilled control. Each animal was monitored closely until it fully recovery from anesthesia. The food intake, stool and urine output, and behavior of animals were monitored until they were sacrificed^25–27^.

#### Sample preparation

Five randomly selected animals were sacrificed at 2, 6, and 8 weeks after surgery were euthanized with intramuscular injection of Zoletil 50 (50 mg/mL) at 15 mg/kg and later CO_2_ asphyxiation for 10 min. The monolithic blocks were extracted and immediately fixed in 10% formaldehyde for Micro CT, histological and histomorphometrical analysis.

#### Micro-computed tomography scanning of new bone formation

Sample blocks were prepared in formalin, and micro-computed tomography (micro-CT) scanning analyses were performed within 2 weeks using SkyScan 1076 (SKYSCAN, Antwerp, Belgium). After setting the micro-CT images, coronal images of the upper peripheral areas of the defect were saved in the database. To measure the tissue area/bone area, two-dimensional morphological analyses were performed. Thus, binary selections of samples from the morphometric analyses were made according to gray-scale density between units 20 and 80. The morphometric analyses were performed using SkyScan 1076 data-viewer software according to the manufacturer's instructions.

#### Histomorphometric analysis

Sections from all paraffin-embedded tissues were routinely stained with hematoxylin and eosin (H&E) and were simultaneously processed to reduce internal staining variations. The optical images of two mid-sections crossing the center of the calvaria defects were used to perform histomorphometrical analysis. For each histological section, the area occupied by the growing bone was identified and measured at a magnification of 200× using the ImageJ software, developed by the National Institutes of Health (NIH IMAGE, Bethesda, MD, USA). These values were used to calculate the percentage of bone area/tissue area.

#### Statistical analyses

All experiments shown in this study were conducted independently, and the results were presented as means ± standard deviation. Microsoft Excel Professional Plus 2016 (Microsoft Software, Redmond, WA, USA) was used for all quantitative statistical analyses. After the statistical analyses, the differences among the groups were compared and considered significant at *p ≤ 0.05, **p ≤ 0.01, ***p ≤ 0.001, and ****p ≤ 0.0001. The two-tailed Student *t*-test was used to compare between groups, respectively.

## Results

### SEM surface morphological observations

**Figure 1:**
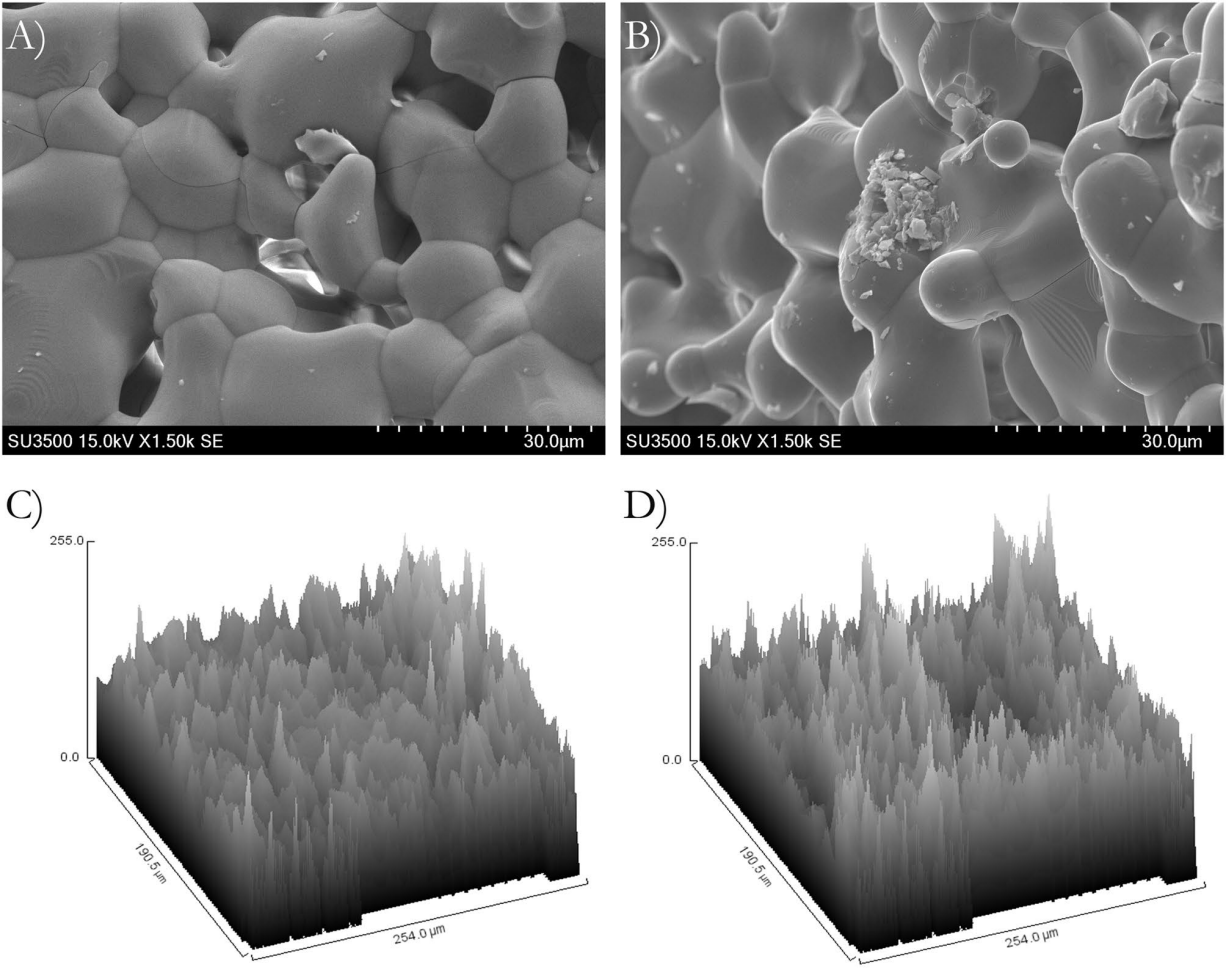
Scanning electron microscope (EX-250 SYSTEM, HORIBA, Kyoto, Japan) of β-TCP surface with (**A**) and without (**B**) GDP-treatment surface treatment, indicating similar surface. Topographic analysis of β-TCP surface with (**C**) and without (**D**) GDP-treatment surface treatment, showing R_a_ slightly smother on GDP-treated β-TCP surface.

The GDP-treated β-TCP and nontreated β-TCP surfaces presented similar surface morphology (Fig. 1A,B). SEM results determined the surface morphology of the β-TCP test was not significantly affected by the argon GDP treatment. The GDP-treated β-TCP surface (20% ± 4.38% R_a_) sample resembled that of the nontreated β-TCP sample (24% ± 4.93% R_a_), but with slightly smoother rough surfaces within the structures (Fig. 1C,D). Furthermore, the number of β-TCP macro and micro particles measuring < 7 μm that were homogeneously distributed on a larger particle surface decreased after GDP treatment (Fig. 1A,B).

### EDS analysis

**Figure 2:**
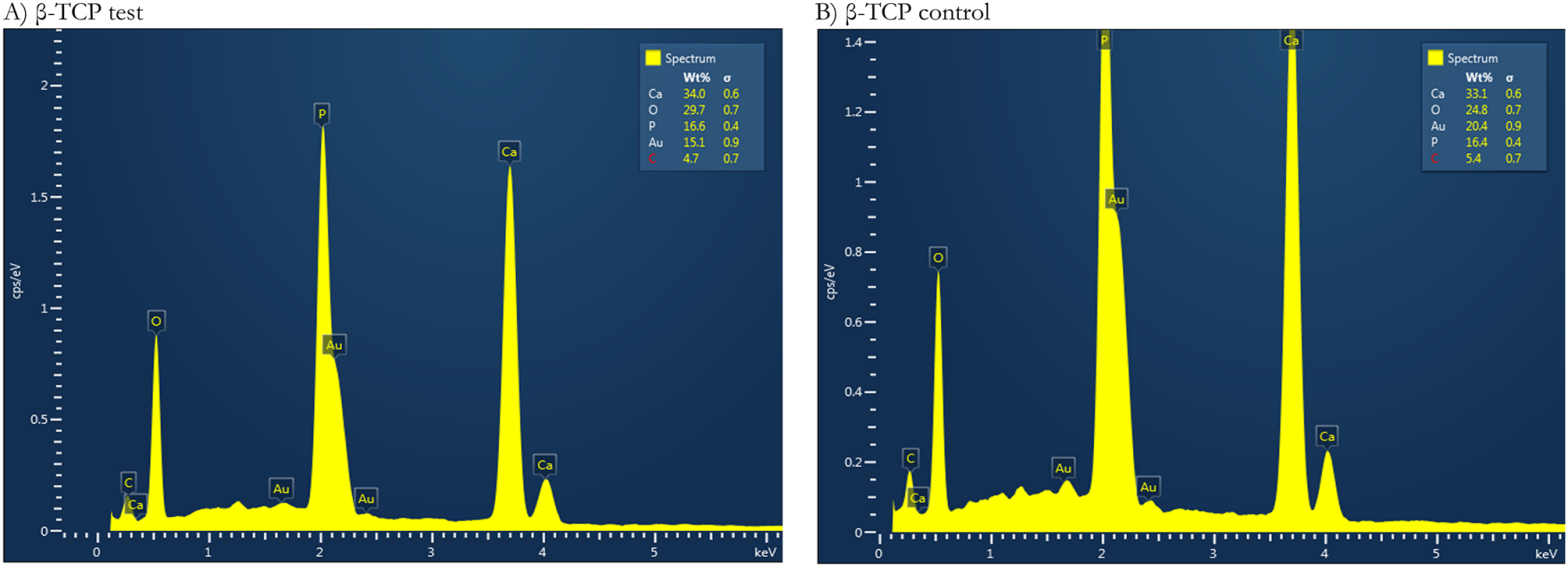
Energy-dispersive spectra (EX-250 SYSTEM, HORIBA, Kyoto, Japan). (**A**) β-TCP test and (**B**) β-TCP control samples.

The concentrations of elements were the same in the two β-TCP samples in the EDS results. More precisely, the β-TCP test specimen contained 34 wt% calcium, 29.7 wt% oxygen, 16.6 wt% phosphorus, 15.1 wt% gold, and 4.7 wt% carbon. By comparison, the elemental concentrations in the β-TCP control specimen were similar to those in the β-TCP test specimen, with 33.1 wt% calcium, 24.8 wt% oxygen, 16.4 wt% phosphorus, 20.4 wt% gold, and 5.4 wt% carbon. β-TCP test particles and β-TCP control had 2.05 and 2.02 Ca/P ratios respectively, which are higher than the human hydroxyapatite value (Table 2; Fig. 2).

**Table 2:** Results of the elemental analysis by energy-dispersive spectrometry.

Element	β-TCP test		β-TCP control	
	Weight%	*σ*	Weight%	*σ*
Ca	34.0	0.6	33.1	0.6
O	29.7	0.7	24.8	0.7
P	16.6	0.4	16.4	0.4
Au	15.1	0.9	20.4	0.9
C	4.7	0.7	5.4	0.7
Total	100	—	100	—

### X-ray photoelectron spectroscopy

**Figure 3:**
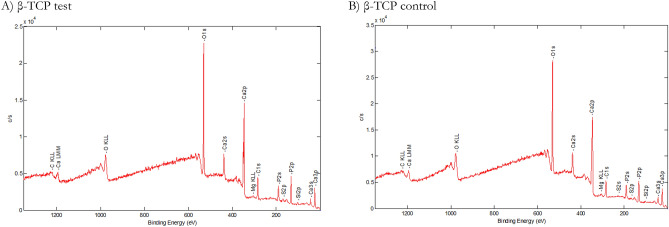
XPS spectra were used to determine the atomic compositions (%). β-TCP test (**A**) and β-TCP control (**B**) specimens show Ca, P, and O. Small amounts of contaminants such as C were present in both β-TCP samples.

The XPS analysis results of the surface chemistry and atomic concentrations of the specimens appear in Table 3. The mean (C1s) values of the surfaces were 18.85% ± 0.20% for the β-TCP test sample and 16.97% ± 0.21% for the β-TCP control sample. Moreover, the (O1s) values for the β-TCP test were 50.60% ± 0.54%, and β-TCP control 53.11% ± 0.66%, respectively. The (P2p) value was 11.63% ± 0.25% in the β-TCP control sample and 11.33% ± 0.09% in the β-TCP test sample. The mean (Ca2p) values were 15.48% ± 0.23% in the β-TCP test sample and 16.55% ± 0.24% in the β-TCP control sample (Table 3; Fig. 3).

**Table 3:** XPS analyses (%).

	C1s	O1s	Si2p	P2p	P2s	S2p	Ca2p	Ca2s
β-TCP test	18.85 ± 0.20	50.60 ± 0.54	2.138 ± 0.04	11.33 ± 0.09	12.32 ± 0.01	0.99 ± 0.01	15.48 ± 0.23	16.02 ± 0.14
β-TCP control	16.97 ± 0.21	53.11 ± 0.66	1.53 ± 0.46	11.63 ± 0.25	10.36 ± 0.01	0.58 ± 0.00	16.55 ± 0.24	16.90 ± 0.13

### XRD analyses

**Figure 4:**
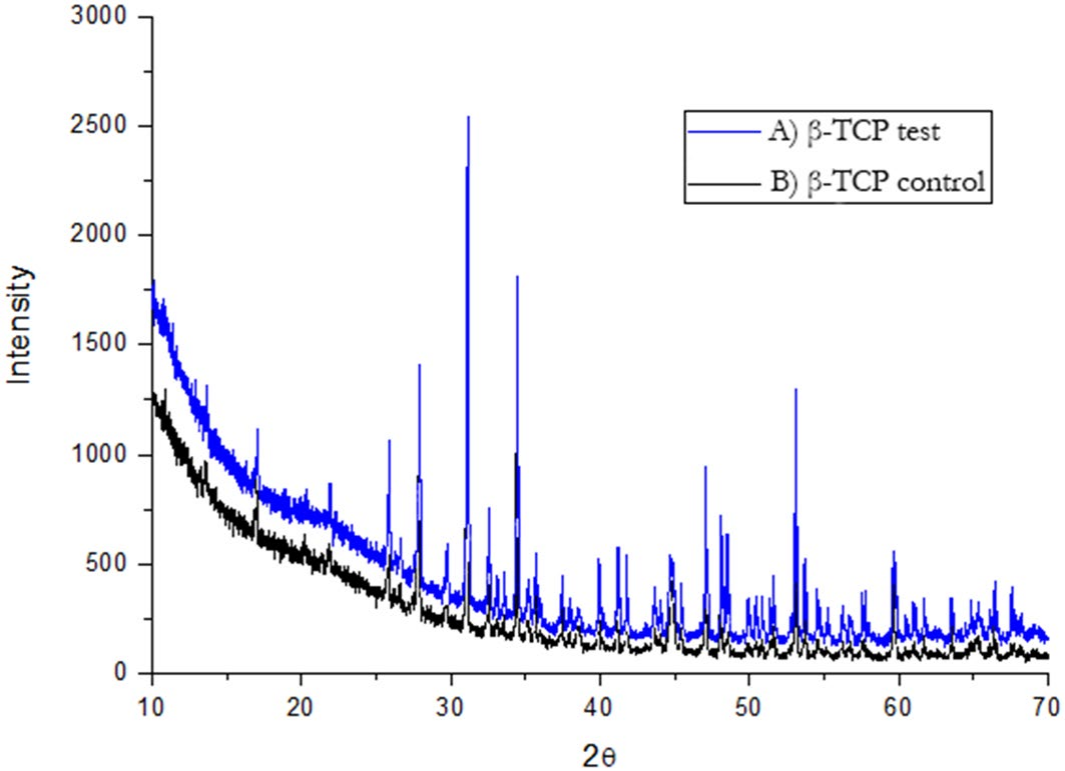
X-ray diffraction patterns (PERKIN-ELMER PHI ESCA 5500 SYSTEM). β-TCP test and β-TCP control samples. The dominant crystalline phases in both materials generate the same intense sharp peaks characteristics of β-TCP (black triangles).

XRD measurements revealed the highly crystalline characteristic of β-TCP in general, as particle grafts before and after GDP treatment, presented similar sharp peak patterns. The β-TCP in both materials is responsible for the higher-intensity peaks (Fig. 4).

### FTIR characterization

**Figure 5:**
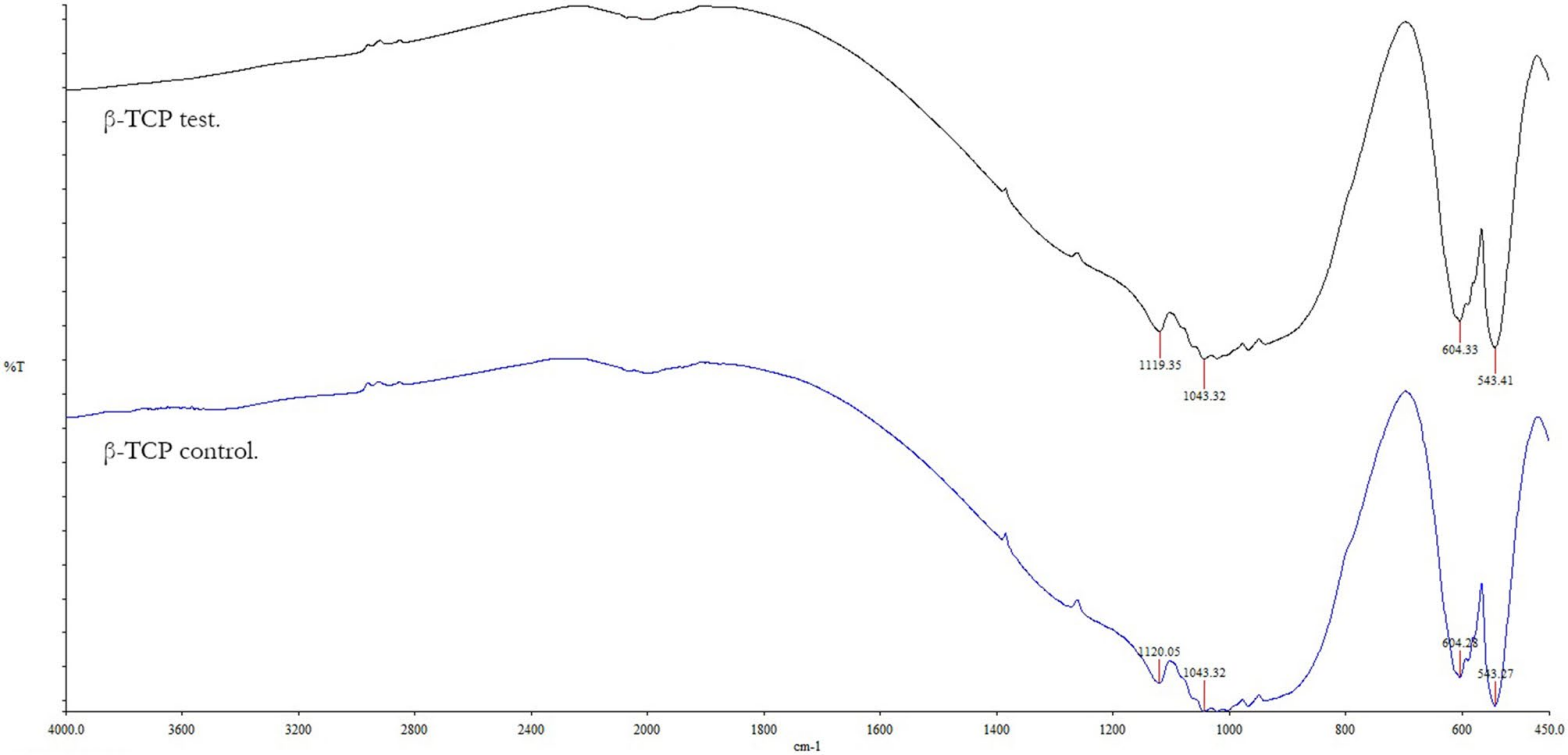
Fourier transform infrared (FTIR) spectroscopy characterization (NICOLET iS50, Thermo Scientific, Madison, USA). FTIR spectra of both β-TCP test and β-TCP control have the same absorption peaks, corresponding to phosphate band peaks.

β-TCP test and β-TCP control particles revealed FTIR spectra results to agree with the XRD analysis. In terms of characteristics, there were no other types of crystalline phase in all samples in addition to the apatite. Similar pronounced peaks close to 470–700 cm^−1^ were found in both β-TCP test and β-TCP control particles^28^. Furthermore, 543, 604, 1043, and 1120 cm^−1^ absorption band peaks belong to phosphatase peaks (Fig. 5).

### Cell proliferation assessment

**Figure 6:**
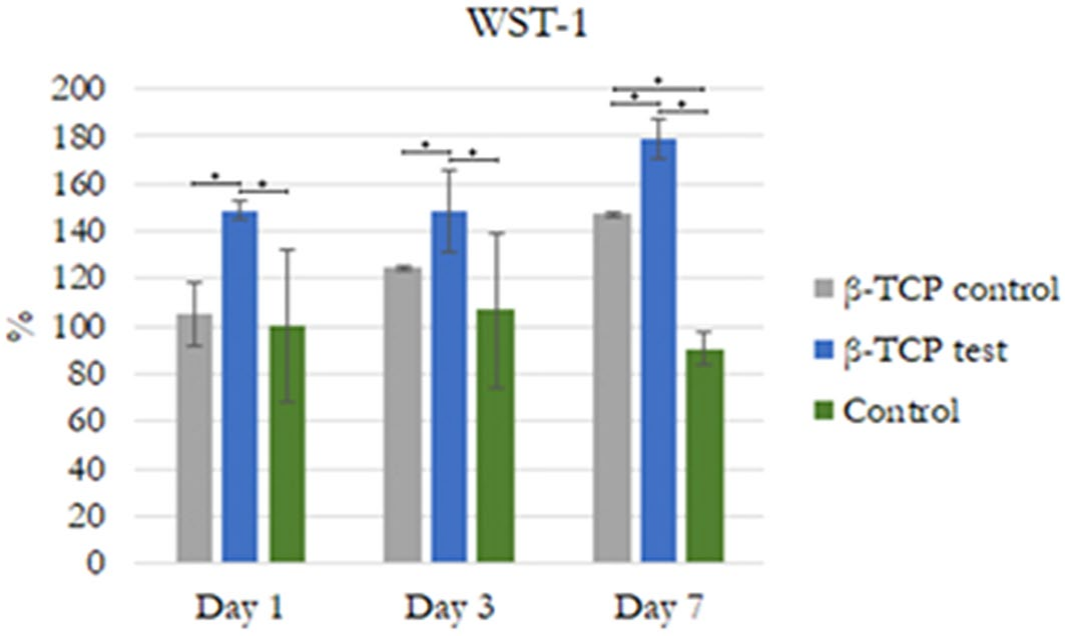
Cell proliferation. WST-1 on days 1, 3, and 7. Statistically significant differences are indicated by *P < 0.05.

The proliferation of hMSCs cultivated in media with GDP-treated β-TCP particles was 106.09%, 132.26%, and 198.52% at days 1, 3, and 7, respectively. At the same time, hMSCs proliferation on the nontreated β-TCP was 100%, 116.73%, and 182.84% at days 1, 3, and 7, respectively. hMSCs presented spreading attachment on the GDP-treated β-TCP surfaces, leading to improved cell proliferation, which were compared with nontreated β-TCP surfaces at day 7 (Fig. 6).

### Cell morphology

**Figure 7:**
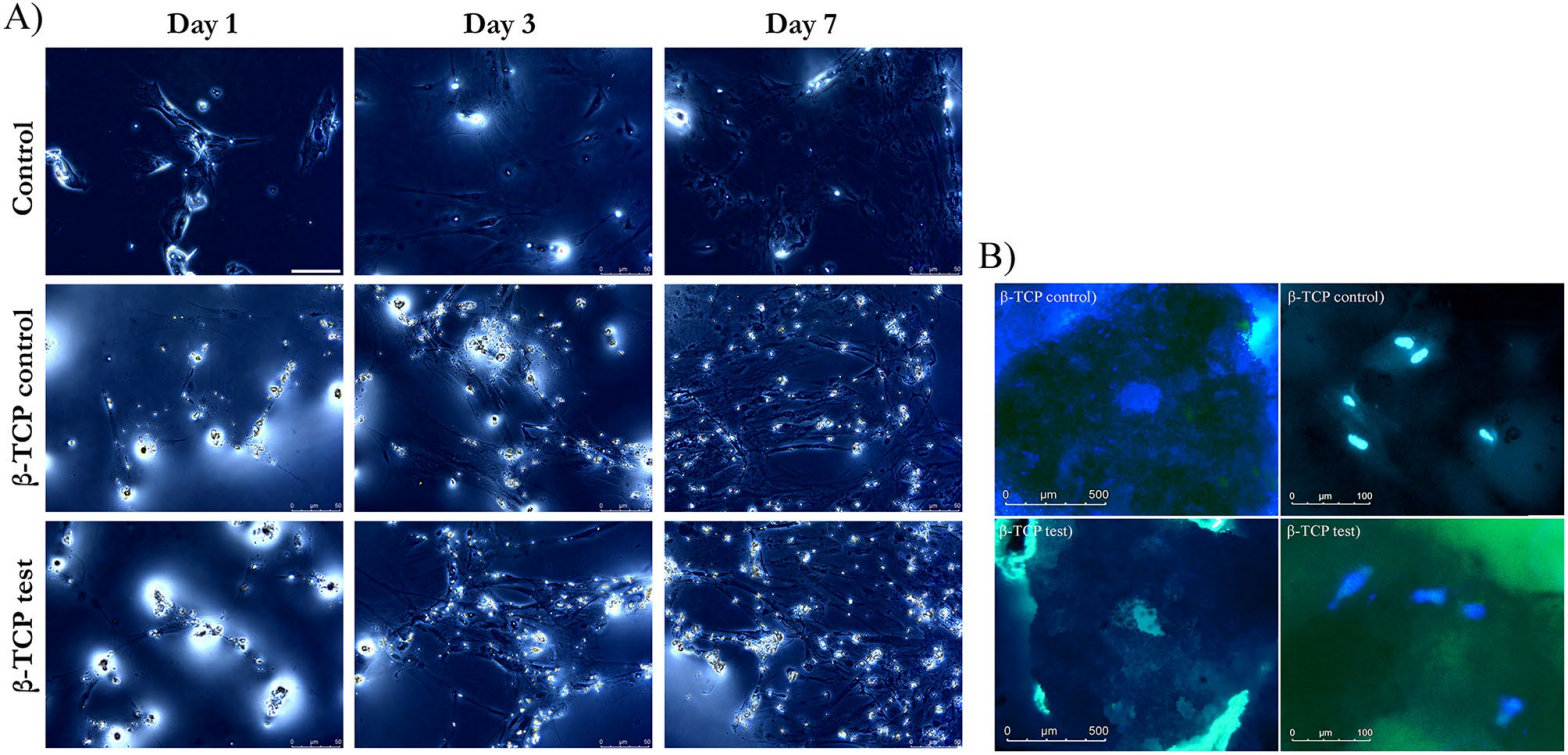
(**A**) Changes in hMSCs morphology at 1, 3, and 7 days. Day 1: Scarce spindle-shaped cells growing in the different media. Day 3: Cells with a more extended morphology had proliferated. Day 7: Cells showing a stage of development with filopodial extensions had proliferated. Magnification 40×. (**B**) 3D Fluorescent imaging with DAPI- phalloidin after 24 h at 20× and 40x.

Morphological analysis via phalloidin/DAPI immunofluorescence staining of hMSCs revealed healthy growth and spreading on β-TCP control and GDP-treated β-TCP test. Most of the cells had a cytoplastic spindle shape and extended morphology with filopodial extensions. Cells spread and proliferated, making possible the formation of relatively thin continuous monolayers at 1, 3, and 7 days within some small particles of the β-TCP control and β-TCP test present within the media in 2D cultured and same results after 24 h in 3D culture (Fig. 7).

### ALP assay

**Figure 8:**
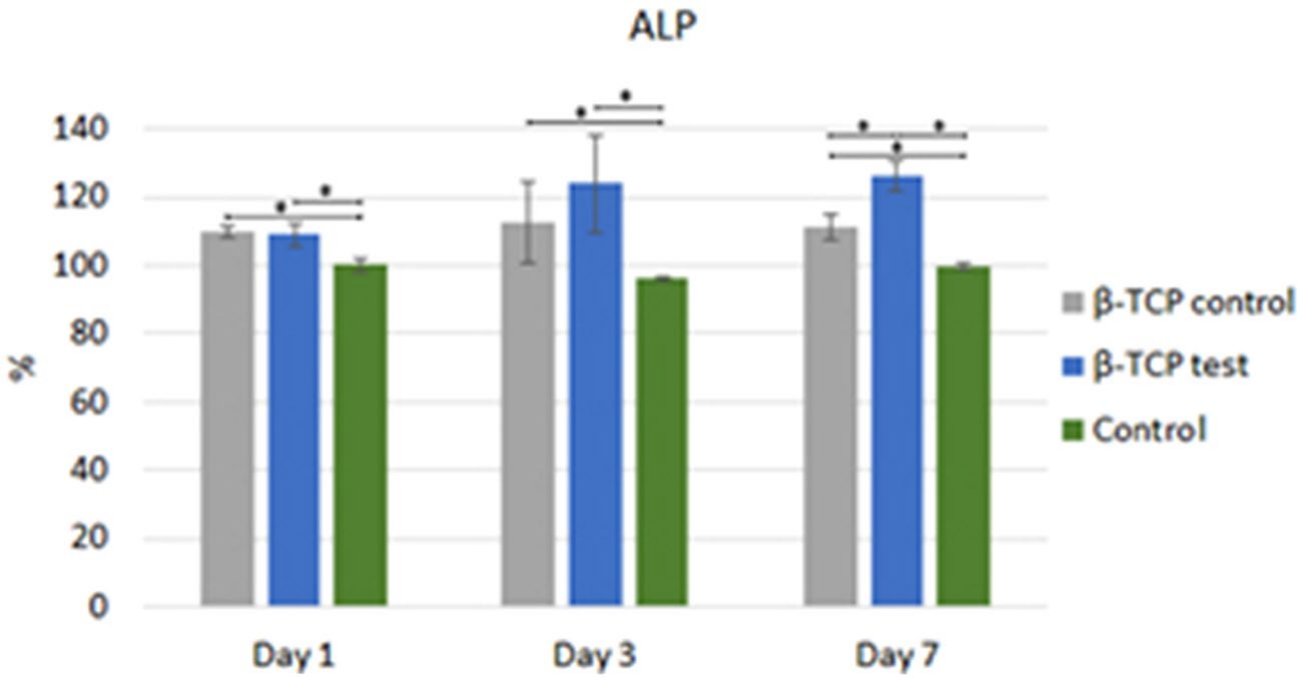
ALP analysis. Statistically significant difference indicated by *P < 0.05.

With 108.85% ± 3.68% at 1 day, at 3 days with 123.92% ± 14.45% and 126.08% ± 4.67% after 7 days, hMSCs cultivated with GDP-treated β-TCP presented higher ALP activity after 3 days of analysis than DMEM-β-TCP control and control cultivated hMSCs. DMEM- β-TCP control cultivated hMSCs at days 1, 3, and 7 had 109.81% ± 1.90%, 112.44% ± 12.06%, 111.24% ± 4% more ALP activity than control hMSCs, which only had 100% ± 2.07%, 96.17% ± 0.72% and 99.52% ± 1.1% of ALP activity at 1, 3, and 7 days respectively (P < 0.05; Fig. 8).

### Real-time polymerase chain reaction

**Figure 9:**
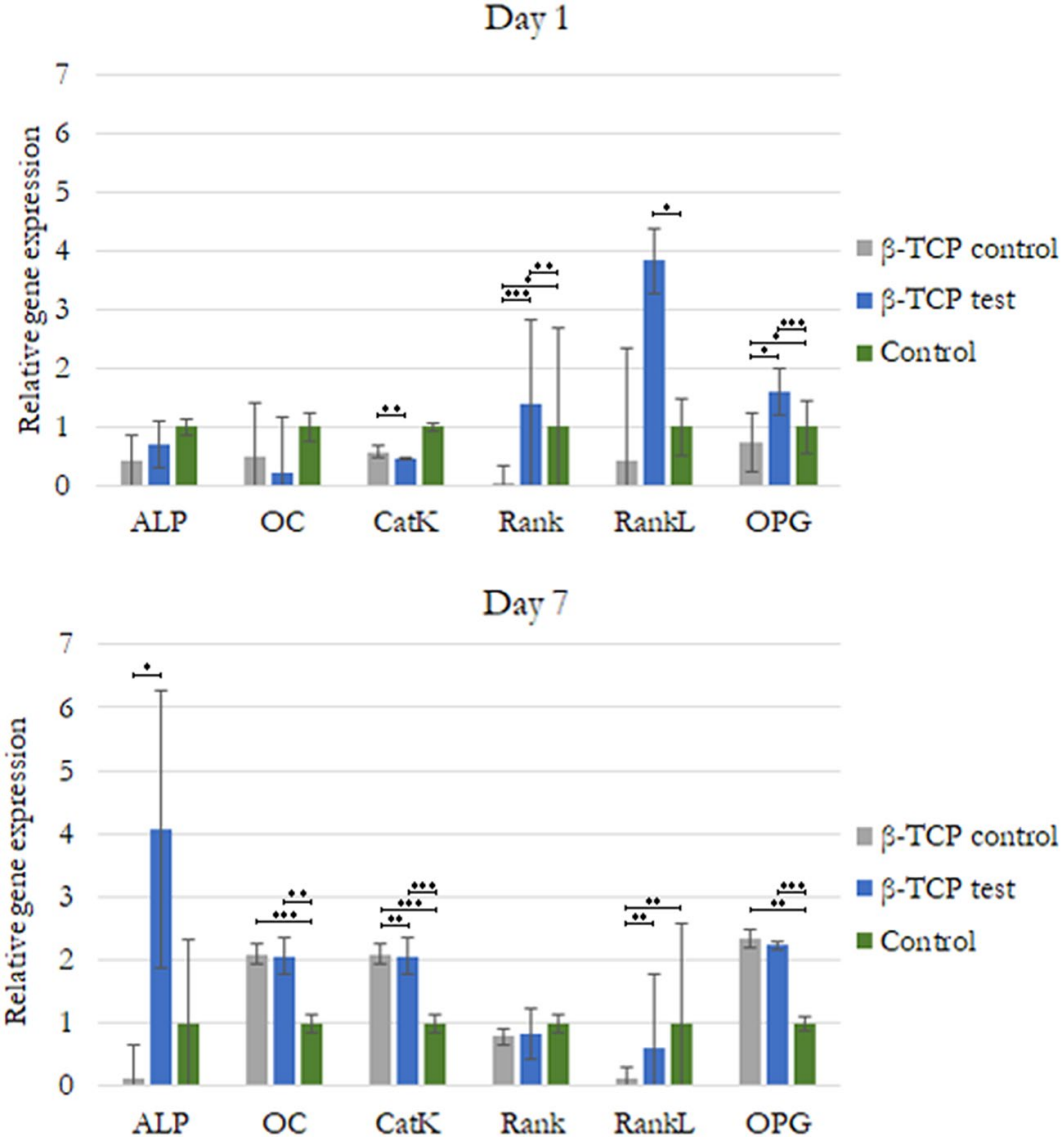
Relative gene expression of hMSCs indicating mainly osteogenic differentiation. Statistically significant difference indicated by *P < 0.05, **P < 0.01, ***P < 0.001.

We analyzed gene expression related to the osteogenic differentiation and function of the hMSCs. RNA was isolated separately from both the cells cultured in DMEM media mixed with β-TCP or β-TCP plasma treated. After a culture of 7 days, there was a significant difference in the expression of crucial osteoclast gene marker RankL and about two times CatK relative gene expression of hMSCs cultured in DMEM-β-TCP control and β-TCP plasma treated over cells cultured in DMEM only. There were also differences in the expression of important genes related to osteoblastic differentiation, such as ALP, Rank, Osteoprotegerin (OPG), and osteocalcin (OC; Fig. 8). ALP was expressed differently on cells cultured with β-TCP plasma-treated media: approximately four times higher relative gene expression over control and β-TCP control cells. However, after 7 days, the expressions of OC and OPG were similar between cells cultured in DMEM media mixed with β-TCP or β-TCP plasma treated, but both values were similarly significantly higher than those of control cells (Fig. 9).

### Micro-CT new bone regeneration

**Figure 10:**
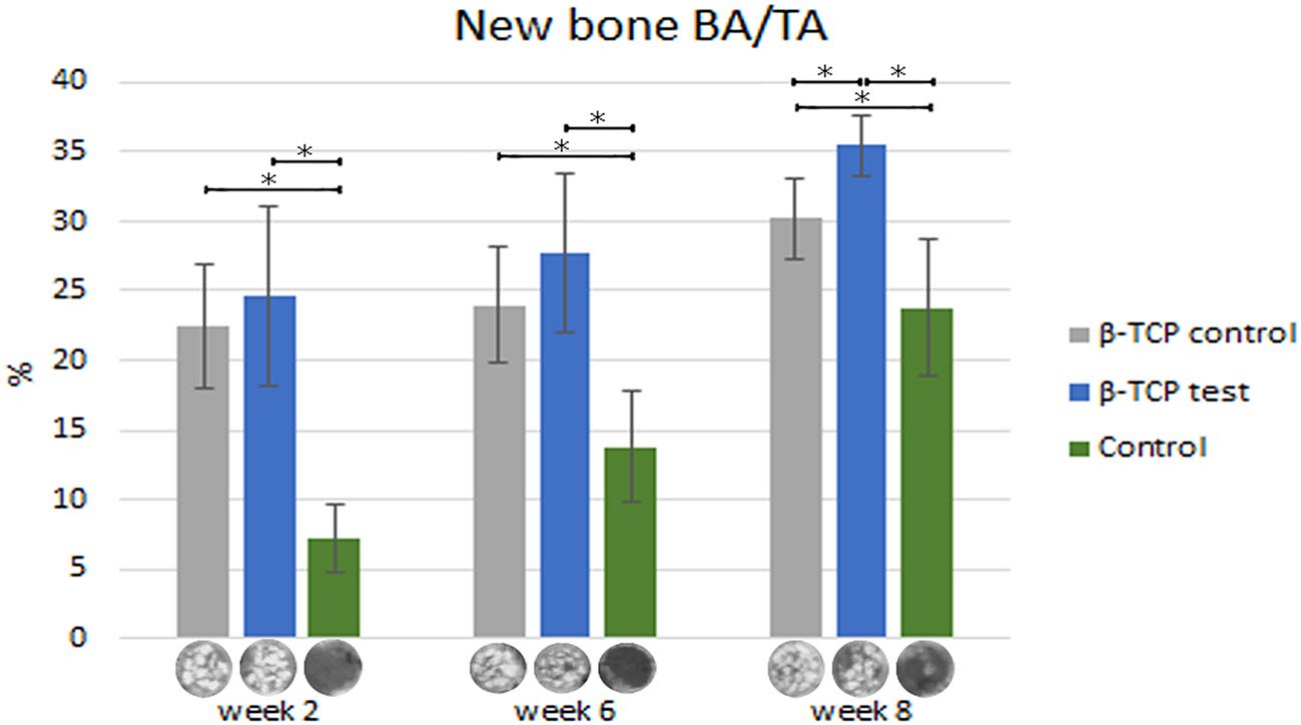
Micro CT new bone formation (SKYSCAN 1076, Antwerp, Belgium). Statistically significant difference was indicated by * (*P* < 0.05).

New bone formation at week 2 calvarial defects filled with β-TCP control, β-TCP test, and control regenerated 22.45% ± 4.5% and 24.64% ± 6.4%, respectively, was statistically significant superior to the 7.16% ± 2.46% new bone regeneration in the control group (*P* < 0.05, Table 4; Fig. 10).

**Table 4:** New bone formation was observed on micro-CT scan.

	Week 2	Week 6	Week 8
β-TCP control	22.45 ± 4.52	23.99 ± 4.13	30.19 ± 2.85
β-TCP test	24.64 ± 6.38	27.72 ± 5.73	35.51 ± 2.21
Control	7.16 ± 2.46	13.81 ± 4.05	23.81 ± 4.83

The β-TCP test at week 6 had 27.72% ± 5.74% new bone regeneration, which was significantly higher than 23.99% ± 4.13% β-TCP control and control groups (13.81% ± 4.05%). A statistically significant difference was found between both β-TCP groups (*P* < 0.05, Table 4; Fig. 10).

At 8 weeks, a similar tendency to the previous weeks, the β-TCP test regenerated 35.51% ± 2.21% higher new bone than the other two groups (*P* < 0.05). The β-TCP control (30.19% ± 2.85%) followed by the control group (23.81% ± 4.83%) formed the least amount of new bone (*P* < 0.05, Table 4; Fig. 10).

### Histomorphometric analysis

**Figure 11:**
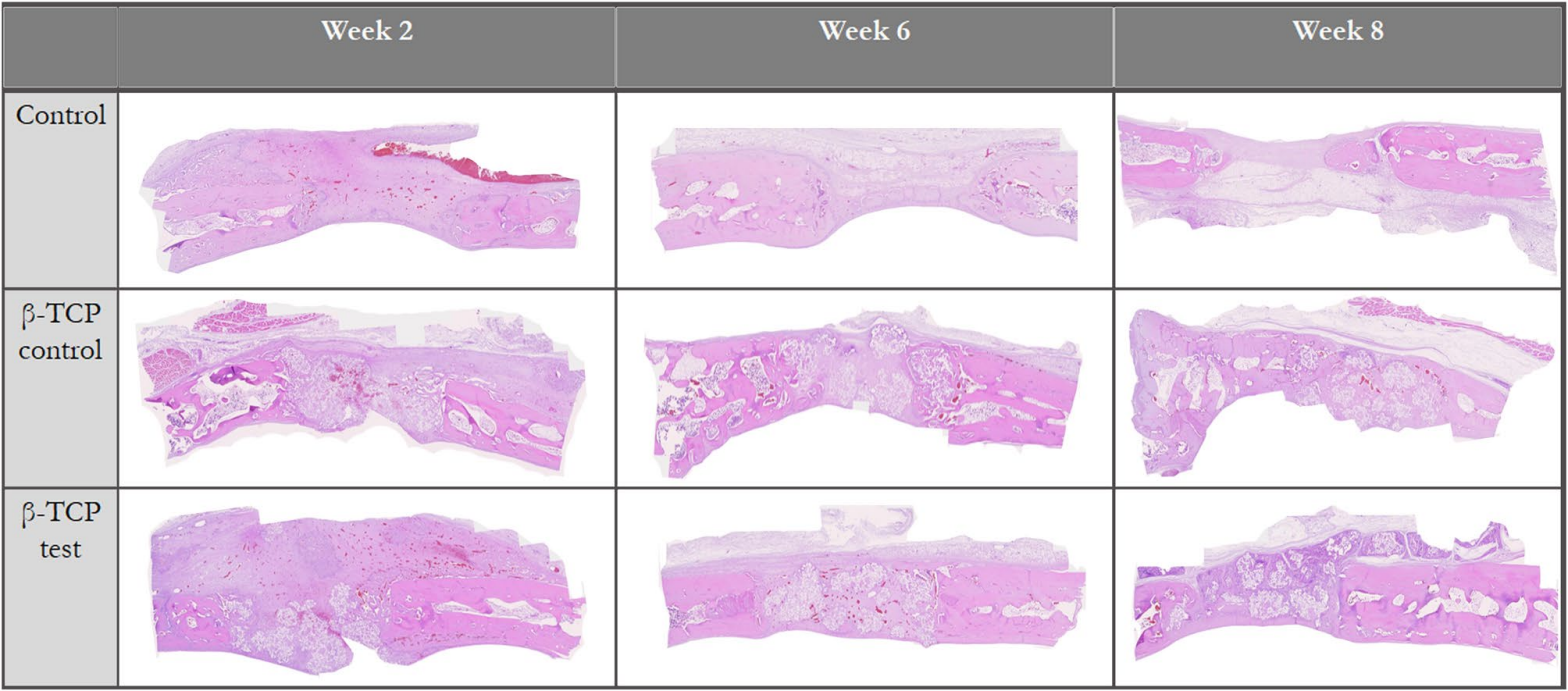
Histologic new bone formation, 40×.

In the second week, it was visible in all the groups predominantly granulation tissue with slightly woven bone surrounded by inflammatory cells and osteoblasts. In the defects in which both types of β-TCP were used, direct contact between the bone and particles was minimum. Immature bone was mainly found at the defect’s borders, with β-TCP particles serving as scaffolds for the newly regenerated woven bone. The control group regenerated 9.91% ± 3.24% new bone, close to the 9.11% ± 5.88% of β-TCP control, Though, the β-TCP test had the statistically significantly highest bone regeneration with 20.28% ± 9.85% (*P* < 0.05, Table 5; Fig. 11).

**Table 5:** Histologic new bone formation bone area/tissue area (%).

	Week 2	Week 6	Week 8
β-TCP control	9.51 ± 5.88	25.46 ± 14.03	22.42 ± 7.12
β-TCP test	20.28 ± 9.85	18.59 ± 7.56	29.67 ± 8.95
Control	9.91 ± 3.24	14.36 ± 8.48	21.71 ± 4.64

Higher new bone regeneration on the surface of some β-TCP test and control particles was observed at 6 weeks. Biomaterial granules with more advanced osteogenic activity than previous weeks was observed on the defect borders; areas where β-TCP test and control granules had more new bone within the particles. In contrast to previous weeks, β-TCP control generated 25.46% ± 14.03% more new bone, which was only statistically significantly different from control group (*P* < 0.05; control group: 14.36% ± 8.48% new bone). Also, the β-TCP test produced 18.59% ± 7.56% new bone, higher than the control, but with no statistically significant difference (*P* < 0.05, Table 5; Fig. 11).

Histology slides demonstrated resorption of the grafts' biomaterials at week 8 with similar new bone formation tendency as in week 2. The β-TCP test (29.67% ± 8.95%) had the highest new bone formation (*P* < 0.05). This new bone was mature in some areas and differentiated from control defects because of graft particles that created a bridge from the defect's border to the center. The proportion of new bone formation in the β-TCP control group (22.42% ± 7.13%) was like that of the control defects (21.71% ± 4.64%; Table 5; Fig. 11).

## Discussion

The β-TCP control samples used in the present work were identical to those used in different in vivo and in vitro clinical studies^2–5,29^, including a 10-year retrospective study that demonstrated reduced crestal bone loss around implants placed immediately after tooth extraction and grafted with β-TCP in extraction sockets^30^. In this previous study, 61 implants were evaluated in which immediate implant was made in fresh extraction sockets with β-TCP placement. The authors showed that β-TCP as a grafting material in immediate implant placement resulted in less bone loss around the implants in 72.1%^30^. Another prospective, multicenter randomized clinical trial evaluated histologic and histomorphometric results between autogenous bone, which is still considered the gold standard, and β-TCP control to avoid donor-site morbidity during bilateral sinus elevation. The study revealed that β-TCP control is a satisfactory graft material, even in the absence of autogenous bone^31^.

Previous studies have demonstrated that TCP surfaces can contain intrinsic impurities present in the starting material^32^. Previous guided tissue regeneration studies have attempted to improve β-TCP qualities^33^, including placing importance on TCP bioceramics surface cleaning for better performance. Four main effects of reactive gas surface treatment include surface cleaning, ablation or etching, surface chemical functionalization, and cross-linking. Each of the effects is present to some degree in a reactive gas process; the extent and degree of the effects depend on the process, energy and frequency, gas chemistry, reactor design, and operating parameters^34^. The GDP treatment on biomaterials can affect the mechanical and biological properties of ceramics during long-term evaluation^35^. Thus, β-TCP biocompatibility, bioactivity, and osteoconductivity characteristics in the present study were improved through surface treatment with argon GDP at 80 W, 13.56 MHz, 100 mTorr, for 15 min, and 10 mm distance of argon plasma to graft granules.

The apatite crystals and organic matrix occupied approximately one- and three-quarters of the bone volume, respectively, which were calculated using bone composition and the densities of the components^36^. These proportions show the importance of the physical and chemical characterization of β-TCP. Based on the SEM results (Fig. 1), the GDP-treated β-TCP surface (20 ± 4.38 R_a_) sample had a slightly less roughness than the nontreated β-TCP sample (24 ± 4.93 R_a_), which is typical for TCP sintered at temperatures of at least 900 °C and can provide good anchorage for cells^37,38^. Seidenstuecker et al. used a Keyence 3D laser scanning microscope (Keyence VK-X210) for measuring a particle’s surface roughness with the same β-TCP biomaterial used in the current study^39^. The surface roughness was 26.08 R_a_, which was similar to that of the nontreated β-TCP sample. In addition, the number of β-TCP macro and micro particles measuring < 7 μm on larger β-TCP particles surfaces decreased. Previous studies have shown that extremely small particles may be immediately resorbed for bone formation^40^. Thus, the removal of these particles after GDP surface treatment is advantageous.

Other results, such as for EDS (Table 2; Fig. 2), demonstrated an increase in oxygen on the β-TCP test surface with 29.7 wt%, whereas the β-TCP control had 24.8 wt%. Despite having used the argon element to bombard the particle graft during the GDP surface treatment, the present study reached similar results to the findings of Roh and his team^41^. In the latter study, the effect of oxygen plasma treatment and the addition of nano-HA and β-TCP on the 3D poly(lactide-co-glycolide) (PLGA) scaffold was investigated. After treatment with oxygen plasma, the hydrophilicity of the scaffold surface increased, and its surface became rough because of the presence of oxygen functional groups on the scaffold surface. Base on their results, oxygen plasma-treated 3D PLGA/nano-HA/β-TCP scaffolds showed higher bioactivity when compared to those of the control^41^. Regardless of these similar outcomes, plasma modification occurs in a particular mechanism for each gas studied, indicating that further studies are necessary^42^.

In addition to EDS, the results of the XRD analyses showed that the β-TCP test material exhibited a higher Ca/P ratio (2.05) than the β-TCP control material (Ca/P = 1.62). Nonetheless, both β-TCP samples had higher ratios than the expected stoichiometric TCP Ca/P ratio of 1.5 reported elsewhere^43^. A possible explanation for the higher Ca/P ratios in both β-TCP materials is the biomaterial sintering process. At the same time, the higher Ca/P ratio of the β-TCP test material compared with that of the control material can be associated only with the plasma sputtering process, which helps eliminate macro and micro particles of < 7 μm in size present on the sample's surface. The same characteristic peaks of dominant crystalline phases for both β-TCP materials are evident in Fig. 4, in which the two different expected stoichiometric TCP phases, α and β, which are less stable but more soluble than HA in aqueous environments, exhibit the same intense sharp peaks with the same peak width, indicating that both materials exhibit the same high crystallinity. In contrast to the poorly crystallized apatites of human bone^36^, both β-TCP samples exhibited greater crystallinity. This characteristic can reduce osteoclastic activity through the saturation of calcium ions that are dissolved continuously on the β-TCP surface^44–46^. This decrease in osteoclastic activity was seen in our study with down-regulation of RankL osteoclast-related gene expressions after 7 days (Fig. 9).

FTIR analysis supported the XRD findings by showing that both materials are apatites with no other crystalline phase, and both particles presented equal sharp peak patterns attributed to phosphatase band peaks. The XPS results indicated that the number of Si ions increased from 1.53% ± 0.46% in the β-TCP control to 2.138% ± 0.04% in the GDP-treated β-TCP. Previously, the addition of Si ions in hydroxyapatite when used as a biomaterial in bone grafting improved the bioactivity and enhancement of bone growth^47^. This finding could explain why WST-1 had statistically significant, superior, and faster cell proliferation, as observed in the β-TCP test sample compared with the β-TCP control sample after 7 days of testing. Using phalloidin/DAPI immunofluorescence staining, the morphological analysis of these cells indicated cells were elongated in appearance when cultured in 2D and 3D. In addition, the presence of Si ions could also explain the ALP results. After 3 days at all time sets, the ALP activity from the hMSCs cultured in β-TCP test media showed a greater increase than that from the hMSCs cultured in β-TCP control media. These results indicate that the plasma surface was able to increase the osteogenic differentiation of hMSCs in vitro.

Mesenchymal stem cells (MSCs) are multipotent cells that act as precursors to osteoblasts. The proliferation and osteogenic differentiation of MSCs are important for the maintenance of osteoblasts. ALP is an early osteoblast marker in the early stage of MSC osteogenic differentiation. However, OC is a gene expressed by mature osteoblasts at later stages of differentiation^48^. Osteoclastogenesis is a multistep process mainly under the control of the essential molecules RankL, RANK, and OPG, which makes the RankL/OPG ratio a major determinant of bone volume and health^49^. To analyze hMSCs osteogenic differentiation and Osteoclastogenesis, we performed quantitative real-time polymerase chain reaction, targeting established markers. We found up-regulation of ALP, OC, and OPG osteogenic genes relative to the control cells in β-TCP control media genes. RANK signaling presented significantly higher relative gene expression in β-TCP test media cell cultured over the other two groups, with a reduction observed after 7 days. These results are in accordance with the findings of Chen et al., who demonstrated that RANK is expressed in bone marrow MSCs and is decreased during osteogenic differentiation. RANK silencing significantly promotes, whereas overexpression suppresses, the osteoblast differentiation of bone marrow MSCs in vitro^50^. This suppression effect on osteoclast differentiation was partially attributed to OPG expression. We found a significant difference in the expression of key osteoclast gene markers RankL and CatK. The former was significantly higher in cells cultured with β-TCP test media than in those cultured with β-TCP control media and media alone and was later reduced to less than that of the control alone. CatK doubled in cells cultured with β-TCP control and β-TCP test media as compared with those cultured with the control media after 7 days. These results indicated that hMSCs mainly indicated osteogenic differentiation expressing a mixture of early and mature osteoblasts after 7 days; at the same time, there was a minimum differentiation in Osteoclastogenesis from some cells. In terms of functionality, the cells cultured with β-TCP test media demonstrated a major feature of osteoblasts, which is to precipitate calcium. Differentiated osteoblasts from the progenitor cells can produce ALP and deposit calcium, which is consistent with bone differentiation^51^. In our study, cells cultured with β-TCP test media after 7 days produced higher ALP than the other cells cultured with β-TCP control and β-TCP test media (*P* < 0.05).

The β-TCP test particle grafts degraded from 2 to 8 weeks of healing, showing similar behavior to that of the β-TCP control. In their study, Sohn et al. evaluated rabbit calvaria defects over a healing period of 2 to 4 weeks for the early phase of healing and of > 8 weeks for the late phase of healing^52^. Thus, our results can be classified as a medium phase of healing. Similar to the findings of Schaller et al.^53^ in rabbit calvarial models, woven bone formation was first observed at 2 weeks of healing in all samples. At 8 weeks, we observed primarily lamellar new bone in the peripheral area. In the central region, both the woven and lamellar bones were seen. The β-TCP test and control particles were in close contact with the new bone but yielded greater defect closure at 8 weeks of healing than control defects did (Figs. 10, 11). However, the new bone formation through osteoconduction was greater for β-TCP test defects than that for β-TCP control (Tables 4, 5).

## Conclusions

The ALP results indicated that the proliferation of hMSCs was substantially higher in argon plasma treated β-TCP than β-TCP control. These results indicate a slight enhancement of the β-TCP received by the argon GDP surface treatment. The in vitro and in vivo tests of the present study showed that the GDP-treated β-TCP surface material has better biocompatibility with cells than the control material, which can possibly lead to more bioactive and osteoconductive material in clinical studies. Further studies involving the connections between the β-TCP test material and bone are needed, including an analysis of angiogenesis, cellular infiltration, attachment through the material's pores, and analysis of calcified tissue deposition over a specified amount of time.

Within the limitations of this study, we conclude that argon GDP surface treatment at 80 W, 13.56 MHz, 100 mTorr, for 15 min is a viable method for removing macro and micro particles of < 7 μm from the surface of β-TCP. It also improves the biocompatibility of β-TCP by increasing the Ca/P ratio and Si ions, while enhancing cell proliferation and osteoblastic differentiation, with a minimum change in surface roughness. And increase bone regeneration through osteoconduction. Still, further studies are necessary.

## References

[1] Basha RY, Doble M (2015). Design of biocomposite materials for bone tissue regeneration. Mater. Sci. Eng. C.

[2] Shalash MA (2013). Evaluation of horizontal ridge augmentation using beta tricalcium phosphate and demineralized bone matrix: a comparative study. J. Clin. Exp. Dent..

[3] Wu J (2015). A pilot clinical study of Class III surgical patients facilitated by improved accelerated osteogenic orthodontic treatments. Angle Orthod..

[4] Kühl S (2013). The influence of bone substitute materials on the bone volume after maxillary sinus augmentation: a microcomputerized tomography study. Clin. Oral. Invest..

[5] Rajan A (2014). Optimized cell survival and seeding efficiency for craniofacial tissue engineering using clinical stem cell therapy. Stem Cells Transl. Med..

[6] Pereira HF, Cengiz IF, Silva FS, Reis RL, Oliveira JM (2020). Scaffolds and coatings for bone regeneration. J. Mater. Sci.: Mater. Med..

[7] Salamanca E (2017). Enhancement of osteoblastic-like cell activity by glow discharge plasma surface modified hydroxyapatite/β-tricalcium phosphate bone substitute. Materials.

[8] Aronsson B-O (1995). Preparation and Characterization of Glow Disharge Modifies Titanium Surfaces.

[9] Aronsson BO, Lausmaa J, Kasemo B (1997). Glow discharge plasma treatment for surface cleaning and modification of metallic biomaterials. J. Biomed. Mater. Res. Part A.

[10] Surmenev RA, Surmeneva MA, Ivanova AA (2014). Significance of calcium phosphate coatings for the enhancement of new bone osteogenesis–A review. Acta Biomater..

[11] Gombotz W, Hoffman A (1987). Gas-discharge techniques for biomaterial modification. CRC Crit. Rev. Biocompat..

[12] Kasemo B, Lausmaa J (1988). Biomaterial and implant surfaces: on the role of cleanliness, contamination, and preparation procedures. J. Biomed. Mater. Res. Part A.

[13] Kasten P (2008). Porosity and pore size of β-tricalcium phosphate scaffold can influence protein production and osteogenic differentiation of human mesenchymal stem cells: an in vitro and in vivo study. Acta Biomater..

[14] Silversmit G, Depla D, Poelman H, Marin GB, De Gryse R (2004). Determination of the V2p XPS binding energies for different vanadium oxidation states (V5+ to V0+). J. Electron. Spectrosc. Relat. Phenom..

[15] Hao J-W, Chen N-D, Fu X-C, Zhang J (2019). Predicting the contents of polysaccharides and its monosugars in Dendrobium huoshanense by partial least squares regression model using attenuated total reflectance Fourier transform infrared spectroscopy. Spectrosc. Lett..

[16] Salamanca E (2020). Porcine collagen-bone composite induced osteoblast differentiation and bone regeneration in vitro and in vivo. Polymers.

[17] Ngamwongsatit P, Banada PP, Panbangred W, Bhunia AK (2008). WST-1-based cell cytotoxicity assay as a substitute for MTT-based assay for rapid detection of toxigenic Bacillus species using CHO cell line. J. Microbiol. Methods.

[18] Bimboim H, Doly J (1979). A rapid alkaline extraction procedure for screening recombinant plasmid DNA. Nucleic Acids Res..

[19] Vogelstein B, Gillespie D (1979). Preparative and analytical purification of DNA from agarose. Proc. Natl. Acad. Sci..

[20] Livak KJ, Schmittgen TD (2001). Analysis of relative gene expression data using real-time quantitative PCR and the 2− ΔΔCT method. Methods.

[21] Sollazzo V (2010). Bio-Oss acts on stem cells derived from peripheral blood. Oman Med. J..

[22] Percie du Sert N (2020). Reporting animal research: Explanation and elaboration for the ARRIVE guidelines 2.0. PLoS Biol..

[23] Cavalcanti SCSXB, Pereira CL, Mazzonetto R, de Moraes M, Moreira RWF (2008). Histological and histomorphometric analyses of calcium phosphate cement in rabbit calvaria. J. Cranio-Maxillofac. Surg..

[24] Behnia H (2013). Bone regeneration with a combination of nanocrystalline hydroxyapatite silica gel, platelet-rich growth factor, and mesenchymal stem cells: a histologic study in rabbit calvaria. Oral. Surg. Oral Med. Oral. Pathol. Oral. Radiol..

[25] Messora MR (2008). Bone healing in critical-size defects treated with platelet-rich plasma: a histologic and histometric study in rat calvaria. J. Periodontal Res..

[26] Donos N (2004). Effect of GBR in combination with deproteinized bovine bone mineral and/or enamel matrix proteins on the healing of critical-size defects. Clin. Oral Implant Res..

[27] Kruse A (2011). Bone regeneration in the presence of a synthetic hydroxyapatite/silica oxide-based and a xenogenic hydroxyapatite-based bone substitute material. Clin. Oral Implant Res..

[28] Fujisawa K (2018). Compositional and histological comparison of carbonate apatite fabricated by dissolution–precipitation reaction and Bio-Oss®. J. Mater. Sci.: Mater. Med..

[29] Harel N, Piek D, Livne S, Palti A, Ormianer Z (2013). 10-year retrospective clinical evaluation of immediately loaded tapered maxillary implants. Int. J. Prosthodont..

[30] Harel N, Moses O, Palti A, Ormianer Z (2013). Long-term results of implants immediately placed into extraction sockets grafted with β-tricalcium phosphate: a retrospective study. J. Oral Maxillofac. Surg..

[31] Szabó G (2005). (2005) A prospective multicenter randomized clinical trial of autogenous bone versus β-tricalcium phosphate graft alone for bilateral sinus elevation: histologic and histomorphometric evaluation. Int. J. Oral Maxillofac. Impl..

[32] Cicek G, Aksoy EA, Durucan C, Hasirci N (2011). Alpha-tricalcium phosphate (α-TCP): solid state synthesis from different calcium precursors and the hydraulic reactivity. J. Mater. Sci. - Mater. Med..

[33] Tian Y (2018). β-tricalcium phosphate composite ceramics with high compressive strength, enhanced osteogenesis and inhibited osteoclastic activities. Colloids Surf., B.

[34] Yasuda H (2004). Luminous Chemical Vapor Deposition and Interface Engineering.

[35] França R, Samani TD, Bayade G, Yahia LH, Sacher E (2014). Nanoscale surface characterization of biphasic calcium phosphate, with comparisons to calcium hydroxyapatite and β-tricalcium phosphate bioceramics. J. Colloid Interface Sci..

[36] Trautz OR (1955). X-ray diffraction of biological and synthetic apatites. Ann. N. Y. Acad. Sci..

[37] LeGeros R, Lin S, Rohanizadeh R, Mijares D, LeGeros J (2003). Biphasic calcium phosphate bioceramics: preparation, properties and applications. J. Mater. Sci.: Mater. Med..

[38] Han Z, Wang C, Shi L (2017). Synthesis and characterization of helium-charged titanium hydride films deposited by direct current magnetron sputtering with mixed gas. Mater. Des..

[39] Seidenstuecker M (2018). 3D powder printed bioglass and β-tricalcium phosphate bone scaffolds. Materials.

[40] Liu J, Kerns DG (2014). Suppl 1: Mechanisms of guided bone regeneration: A review. Open Dent. J..

[41] Roh H-S, Jung S-C, Kook M-S, Kim B-H (2016). In vitro study of 3D PLGA/n-HAp/β-TCP composite scaffolds with etched oxygen plasma surface modification in bone tissue engineering. Appl. Surf. Sci..

[42] Wilson D, Rhodes N, Williams R (2003). Surface modification of a segmented polyetherurethane using a low-powered gas plasma and its influence on the activation of the coagulation system. Biomaterials.

[43] Tahriri M (2018). Biomaterials for Oral and Dental Tissue Engineering.

[44] McLeod K (2006). XPS and bioactivity study of the bisphosphonate pamidronate adsorbed onto plasma sprayed hydroxyapatite coatings. Appl. Surf. Sci..

[45] Xin R, Leng Y, Chen J, Zhang Q (2005). A comparative study of calcium phosphate formation on bioceramics in vitro and in vivo. Biomaterials.

[46] Yamada S, Heymann D, Bouler J-M, Daculsi G (1997). Osteoclastic resorption of calcium phosphate ceramics with different hydroxyapatite/β-tricalcium phosphate ratios. Biomaterials.

[47] Bang L, Ishikawa K, Othman R (2011). Effect of silicon and heat-treatment temperature on the morphology and mechanical properties of silicon-substituted hydroxyapatite. Ceram. Int..

[48] Carvalho A (2018). Femtosecond laser microstructured Alumina toughened Zirconia: A new strategy to improve osteogenic differentiation of hMSCs. Appl. Surf. Sci..

[49] Sharaf-Eldin WE, Abu-Shahba N, Mahmoud M, El-Badri N (2016). The modulatory effects of mesenchymal stem cells on osteoclastogenesis. Stem Cells Int..

[50] Cao X (2018). RANKL-RANK signaling regulates osteoblast differentiation and bone formation. Bone research.

[51] Atala A (2012). Progenitor and Stem Cell Technologies and Therapies.

[52] Yip I, Ma L, Mattheos N, Dard M, Lang NP (2015). Defect healing with various bone substitutes. Clin. Oral Implant Res..

[53] Schaller B (2020). Effects of additional collagen in biphasic calcium phosphates: a study in a rabbit calvaria. Clin. Oral Invest..

